# Spatial multi-omics technologies in gastric cancer: applications and advances

**DOI:** 10.3389/fimmu.2026.1767512

**Published:** 2026-04-14

**Authors:** Qianqian Liu, Haowen Liu, Jing Lv, Jiashuo Li, Guangtan Du, Wensheng Qiu, Shasha Wang

**Affiliations:** 1 Department of Oncology, Affiliated Hospital of Qingdao University, Qingdao, China; 2 Qingdao University College of Medicine, Qingdao University, Qingdao, China

**Keywords:** artificial intelligence, cancer-associated fibroblasts (CAFs), clinical translation, gastric cancer, spatial genomics, spatial metabolomics, spatial proteomics, spatial transcriptomics

## Abstract

Gastric cancer (GC) is plagued by profound intratumoral heterogeneity and a complex tumor microenvironment (TME), which are the core obstacles to precise diagnosis and treatment. Conventional bulk multi-omics technologies average molecular signals across tissues, thus masking cellular heterogeneity; single-cell multi-omics resolves cellular diversity but dissociates cells from their native spatial context, leading to the loss of critical information on intercellular crosstalk and molecular spatial distribution. These limitations result in an incomplete understanding of GC pathogenesis and TME regulatory networks. Spatial multi-omics technologies, integrating genomics, transcriptomics, proteomics, and metabolomics with high-resolution spatial localization, address these key scientific problems by preserving the native tissue architecture and elucidating the spatiotemporal dynamics of molecular and cellular events in GC. This review systematically synthesizes the latest advances in the application of four major spatial multi-omics modalities in GC research over the past 15 years, with a critical evaluation of the technical performance, methodological shortcomings, and clinical translation potential of existing studies. Unlike previous reviews that only summarize research findings, this work uniquely integrates technical principles, mechanistic discoveries, and clinical translation of spatial multi-omics in GC, deeply analyzes the practical barriers to clinical application, and systematically elaborates the integration of spatial multi-omics with artificial intelligence (AI). We also identify unresolved challenges in the field and propose future development directions, providing a comprehensive and in-depth reference for the advancement of GC precision medicine based on spatial multi-omics.

## 1 Introduction

Gastric cancer ranks as the fifth most common cancer globally, accounting for over 1 million new cases diagnosed annually (1). In China, it represents a major public health burden, ranking fifth in incidence and third in mortality (2). Despite considerable progress in diagnostic and therapeutic strategies in recent years, therapeutic outcomes remain suboptimal due to the disease’s complex heterogeneity. Despite advances in diagnostic and therapeutic strategies, the overall prognosis of GC remains unsatisfactory, fundamentally due to its profound intratumoral heterogeneity and intricate TME intercellular crosstalk. Conventional multi-omics technologies have provided important insights into GC pathogenesis and biomarker discovery (3), but they suffer from inherent limitations: bulk transcriptomics/proteomics average molecular signals across the entire tissue, failing to distinguish the unique molecular characteristics of different cell subpopulations; single-cell sequencing resolves cellular heterogeneity but loses the spatial context of cell distribution and intercellular interactions, making it impossible to reveal the spatial regulatory network of the TME. These technical flaws lead to a fragmented understanding of GC, hindering the development of accurate diagnostic biomarkers and personalized therapeutic strategies.

Spatial multi-omics, an emerging interdisciplinary technology that combines high-throughput molecular profiling with *in situ* spatial localization, has emerged as a powerful tool to break through the above bottlenecks. This technology covers four core modalities—spatial genomics, spatial transcriptomics, spatial proteomics, and spatial metabolomics (4) —each with distinct technical principles, resolution characteristics, and application scenarios (**Table 1**). By preserving the native tissue architecture, spatial multi-omics can elucidate the spatiotemporal distribution of genomic variations, gene expression, protein localization, and metabolite abundance in GC tissues, and further reveal the molecular mechanisms of tumor initiation, progression, drug resistance, and TME regulation at the spatial level (5). Since *Nature* identified spatial multi-omics as one of the seven disruptive technologies of 2022 (6), its application in GC research has developed rapidly, yielding a series of important discoveries, such as novel gastric stem cell markers, subtype-specific cancer-associated fibroblast (CAF) subsets, and metabolic subtypes associated with targeted therapy response.

**Figure 1:**
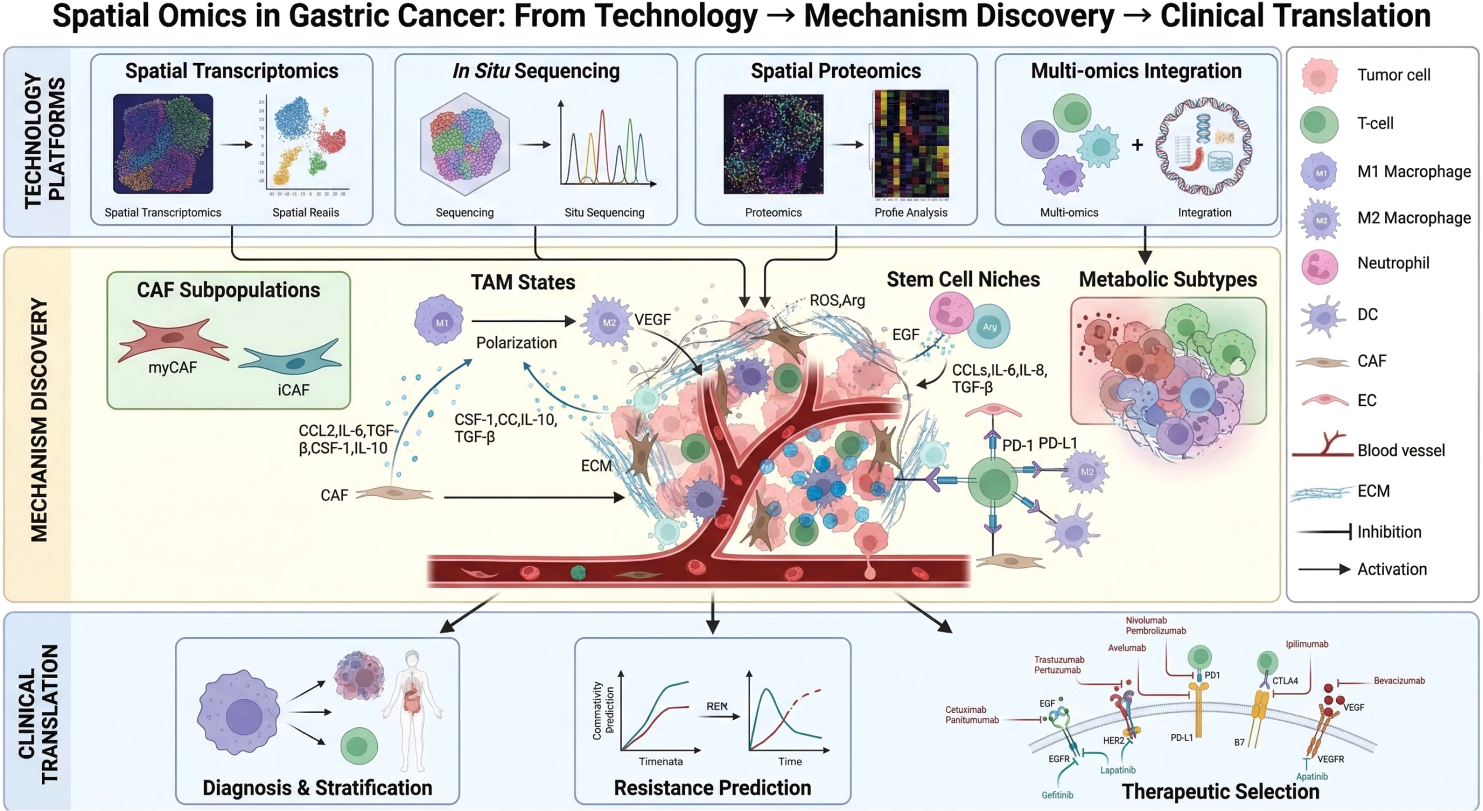
Spatial omics in gastric cancer: from technology - mechanism discovery - clinical translation. This conceptual schematic illustrates the full translational pipeline of spatial omics in gastric cancer (GC) research, organized into three interconnected modules: 1. Technology Platforms: Depicts four core modalities–spatial transcriptomics, in situ sequencing, spatial proteomics, and multi-omics integration–highlighting their technical workflows for in situ molecular profiling. 2. Mechanism Discovery: Centers on the GC tumor microenvironment (TME), showcasing key findings including CAF subpopulations, TAM polarization dynamics, stem cell niches, and metabolic subtypes, which reveal spatial heterogeneity and intercellular crosstalk. 3. Clinical Translation: Translates mechanistic insights into clinical applications: diagnosis & risk stratification, treatment resistance prediction, and precision therapeutic selection (e.g., immunotherapies, targeted agents). This figure demonstrates the unique value of spatial omics in decoding GC heterogeneity and advancing precision medicine. Created with https://www.biorender.com. CAF, cancer-associated fibroblast; myCAF, myofibroblastic CAF; iCAF, inflammatory CAF; TAM, tumor-associated macrophage; M1, classically activated macrophage; M2, alternatively activated macrophage; ECM, extracellular matrix; EC, endothelial cell; DC, dendritic cell; VEGF, vascular endothelial growth factor; EGF, epidermal growth factor; TGF-β, transforming growth factor-β; CCL, chemokine (C-C motif) ligand; IL, interleukin; CSF, colony-stimulating factor; ROS, reactive oxygen species; Arg, arginine; PD-1, programmed cell death protein 1; PD-L1, programmed cell death ligand 1; CTLA4, cytotoxic T-lymphocyte-associated protein 4; HER2, human epidermal growth factor receptor 2; EGFR, epidermal growth factor receptor; VEGFR, vascular endothelial growth factor receptor; REH, resistance-evading hub.

However, the current research on spatial multi-omics in GC is still in the exploratory stage, with obvious limitations: uneven technical development across modalities, small sample sizes in most studies, insufficient external validation of findings, and a low clinical translation rate. Most existing reviews only summarize the technical applications and research findings of spatial multi-omics in GC, lacking a critical evaluation of the shortcomings of existing studies and an in-depth discussion of the practical barriers to clinical translation. In addition, the integration of spatial multi-omics with AI, a key direction for future development, has not been systematically elaborated. Given these gaps, this review aims to (1): systematically sort out the technical characteristics, advantages, and limitations of four major spatial multi-omics modalities (2); critically evaluate the latest research findings in GC and their biological significance; (3) add a dedicated section on the integration of spatial multi-omics and AI with concrete methodological examples; (4) comprehensively analyze the barriers to clinical translation of spatial multi-omics in GC and propose targeted solutions; (5) construct a novel conceptual figure reflecting the whole process of “spatial multi-omics technology → GC mechanistic discovery → clinical translation”. This review is expected to fill the existing research gaps and provide a systematic reference for researchers in the field of GC and spatial multi-omics, promoting the development and clinical translation of spatial multi-omics technology in GC precision medicine. To systematically present the core logic of this review, we constructed a conceptual schematic (**Figure 1**) that maps the full chain from spatial multi-omics technical platforms (spatial genomics, transcriptomics, proteomics, metabolomics) to mechanistic insights (e.g., CAF subtypes, metabolic reprogramming) and clinical translation (diagnosis, resistance prediction, precision therapy). This figure provides an intuitive overview of how spatial multi-omics deciphers gastric cancer heterogeneity and drives therapeutic innovation.

## 2 Spatial multi-omics technologies: their capabilities and limitations

Spatial multi-omics technologies enable the *in situ* analysis of molecular characteristics at the genomic, transcriptomic, proteomic, and metabolomic levels while preserving the native tissue structure, and each modality has unique technical principles, resolution, and application scope. The core technical parameters, advantages, and limitations of the four major spatial multi-omics modalities are summarized in **Table 1**.

**Table 1:** Comparison of technical parameters.

Modality	Spatial genomics		Spatial transcriptomics		Spatial proteomics		Spatial metabolomics		
Sub-technology	Slide-DNA-seq	IGS	10X Visium/Visium HD	ISH	mIHC	DSP	MALDI-MSI	DESI-MSI	SIMS
Resolution	10 μm	Sub-cellular	55 μm/Single-cell	Sub-cellular	Sub-cellular	200 μm	2–10 μm	50–200 μm	50 nm
Throughput	Medium	Low	High	Low	Medium	Medium	Medium	Medium	Low
Cost	Medium-High	High	Medium	Medium-High	Low-Medium	Medium-High	Medium-High	Medium	High
Advantages	Map genomic clonal heterogeneity; High spatial localization accuracy	Simultaneous genome sequencing and imaging; Intact sample analysis	High-throughput transcriptome profiling; Comprehensive gene expression coverage	Accurate RNA localization; Low detection error rate; Simple operation	Cost-effective; High specificity; Suitable for clinical sample detection	High sensitivity; Regional-specific protein detection; High throughput	Label-free detection; High sensitivity; Minimal sample restrictions	No matrix coating; Real-time detection; Suitable for fresh tissue	Ultra-high resolution; Subcellular metabolite localization
Key Limitations	Low experimental efficiency; Strict sampling constraints	Narrow tissue coverage; Complex experimental operation; High technical threshold	Multicellular resolution (Visium); High cost of single-cell version	Low hybridization efficiency; Limited gene detection range	Signal overlap in multi-labeling; Limited to known protein detection	Low spatial resolution; Dependent on specific panels	Incomplete metabolite database; Demanding sample preparation	Low resolution; Poor quantitative accuracy	Severe sample damage; Complex instrumentation; Difficult data interpretation
Applications	Gastric stem cell genomic analysis; Precancerous lesion genomic profiling	Single-cell level genomic variation detection	TME cell subset identification; Prognostic marker screening	Target gene spatial expression verification	PD-L1/HER2 co-localization analysis; Immune cell infiltration detection	Spatial-specific protein marker screening	Metabolic subtype classification; Drug distribution mapping	*In-vivo* metabolic dynamic detection	Single-cell level metabolic analysis
References	(7–9)		(10–13)		(3, 5, 14, 15)		(16–18)		

### 2.1 Spatial genomics

Spatial genomics investigates genomic sequence variations and three-dimensional structural changes among cells within the context of native tissues. By resolving gene expression and genomic variation while preserving spatial information, this approach significantly enhances our understanding of cellular distribution and function within tissues. Gene expression regulation involves multiple factors, including genetic and environmental influences, making it challenging to establish genotype-phenotype correlations. However, transcriptome analysis can address this challenge (7). Given the heterogeneity of gene expression, single-cell RNA sequencing and spatial transcriptomics techniques enable investigation of cell populations and interactions in intact tissue sections. Spatial genomics encompasses a variety of technical approaches, primarily categorized as follows: Imaging-based technologies (e.g., DNA seqFISH+ and DNA-MerFISH), Sequencing-based platforms (e.g., 10X Genomics Visium, slide-DNA-seq, and spatial ATAC-seq), and those based on *in situ* sequencing and multiple *in situ* hybridization (8). Among these, Slide-DNA-seq detects clonal heterogeneity and spatially map genomic information within tissues, thereby improving tumor detection, localization, diagnostic accuracy, and precise therapy (9). The integration of genomic and spatial technologies—exemplified by *in situ* genome sequencing (IGS)—enables simultaneous sequencing and imaging of genomic information while preserving critical spatial location context.

Spatial genomics technologies present distinct advantages and limitations. Imaging-based techniques (e.g., DNA seqFISH+ and DNA-MerFISH) yield intuitive results and enable simultaneous acquisition of genomic, transcriptomic, and proteomic information. Nevertheless, they involve complex experimental protocols and are challenging to implement in large-scale sample analyses. In contrast, sequencing-based technologies (e.g., 10X Genomics Visium, slide-DNA-seq, and spatial ATAC-seq) offer distinct advantages including high throughput, enhanced resolution, and comprehensive data generation. However, the data generated by these methods is highly complex, large in volume, and relatively expensive to obtain. Regarding *in situ* sequencing and multiplex *in situ* hybridization techniques, their strengths include high sensitivity and excellent preservation pf cellular spatial positioning. These benefits are counterbalanced by limitations such as technically demanding procedures, low throughput, and substantial cost.

### 2.2 Spatial transcriptomics

Spatial transcriptomics provides a powerful framework for the *in situ* mapping and quantitative analysis of genome-wide gene expression while preserving native tissue architecture. Unlike bulk RNA sequencing, this technology preserves spatial context through whole-tissue section analysis, enabling simultaneous investigation of gene expression and tissue architecture, as well as the elucidation of molecular interactions between tissue components and their underlying mechanisms (10).

Spatial transcriptomics techniques are primarily categorized into two types: next-generation sequencing (NGS)-based methods and imaging-based methods. The latter encompasses *in situ* hybridization (ISH) and *in situ* sequencing (ISS) (11). This technology can detect genome-wide gene expression in tissues and provide comprehensive transcriptome profiles. A primary limitation, however, lies in its spatial resolution, which, often remains at a multi-cellular level and infrequently achieves true single-cell resolution (12). Additionally, incomplete data capture may occur, resulting in the loss of gene expression signals from specific tissue compartments (13).

### 2.3 Spatial proteomics

Spatial proteomics leverages subcellular-resolution imaging technologies to systematically map protein localization, protein modification states, and associated mRNA within tissue samples, aiming to reveal how cellular spatial distribution influences disease processes. Although current spatial multi-omics technologies face limitations—such as high costs and lengthy processing times—this emerging field effectively compensates for the inadequate spatial resolution of traditional proteomics and overcomes the limitation that conventional omics approaches struggle to reveal the precise localization and interactions of proteins within tissues. Spatial proteomics has yielded significant insights into diverse research areas including developmental biology and neuroanatomy, while also providing microscopic perspectives on the TME and its relevance to patient prognosis (19).

The continuous advancement of spatial proteomics techniques—including multiplex immunohistochemistry (mIHC), *in situ* mass spectrometry imaging, and spatial transcriptome sequencing—enables researchers to analyze the spatial distribution characteristics and interaction networks at the cellular and even subcellular resolutions. Key methodologies include the following (14): mass spectrometry imaging (e.g., MALDI-MSI) and multiplex fluorescence *in situ* hybridization (mFISH). As a rapidly advancing discipline, spatial proteomics facilitates investigation of protein abundance and localization within cellular and tissue microenvironments. While holding particular promise for cancer research, this technology still has certain limitations: 1. Resolution constraints: Although techniques (e.g., MERFISH and SEQFISH+) achieve higher resolution, their experimental complexity, and incomplete cellular tissue coverage remains challenging (20). 2. Multi-labeling complexity: Simultaneous detection of multiple protein biomarkers increases risks of signal overlap and crosstalk, limiting multiplexing capacity. The requirement for target-specific antibodies or probes further escalates costs (15). In summary, spatial proteomics provides valuable tools for oncology research, though ongoing technological refinement and methodological innovation are needed to address current limitation (3, 5).

### 2.4 Spatial metabolomics

Spatial metabolomics, an emerging molecular imaging discipline, integrates mass spectrometry imaging (MSI) with metabolomics analysis approaches, enabling the qualitative identification, qualitative assessment, and spatial localization of metabolites within tumor tissues (16). The technological foundation resides in advanced MSI techniques, including matrix-assisted laser desorption/ionization mass spectrometry(MALDI), desorption electrospray ionization(DESI), and secondary ion mass spectrometry(SIMS) (4). This approach resolves a critical limitation of conventional metabolomics—the loss of spatial information—thereby facilitating holistic characterization of the tumor immune microenvironment while preserving the precise spatial distribution patterns of metabolites. Given that lipids and sugars serve as fundamental structural and functional biomolecules, spatially resolved investigations (spatial lipidomics and spatial glycomics) hold significant potential for the early discovery of pivotal tumor biomarkers (17).

Furthermore, spatial metabolomics demonstrates superior sensitivity compared to conventional methods, permitting label-free, direct analysis of biological specimens and highly multiplexed metabolite detection, thereby minimizing potential analytical artifacts. By acquiring multidimensional spatial distribution data, researchers can elucidate fundamental disparities in metabolite localization between malignant and adjacent normal tissues. Notable challenges persist, however, including incomplete metabolite spectral databases, technically demanding sample preparation protocols, and computationally intensive data analysis. Future research should prioritize addressing these challenges through strategic integration of multi-omics frameworks and leveraging artificial intelligence-driven computational platforms to unlock the field’s transformative potential.

### 2.5 Integration of spatial multi-omics technologies

The past decade has witnessed the rapid development and widespread application of spatial transcriptomics, spatial proteomics, spatial genomics, and spatial metabolomics across diverse research fields. However, these technologies are typically capture information at a single omics level. Consequently, the integration of heterogeneous spatial multi-omics data has emerged as a critical frontier for future advancement. Spatial multi-omics integration enables the simultaneous analysis of multiple molecular modalities with tissue sections, facilitating systematic investigation of cancer-related molecular alterations to identify biomarkers and therapeutic targets. Prominent examples include: Spatial chromatin-mRNA profiling: Technologies like spatial ATAC&RNA-seq and spatial CUT&Tag RNA-seq concurrently map chromatin accessibility and mRNA expression, revealing gene regulatory mechanisms (4). Spatial metabolomics: Mass spectrometry imaging provides comprehensive visualization of tumor metabolic reprogramming and intercellular interactions, elucidating the spatial attributes of metabolites within tumor tissues (21), and enabling screening of tumor-associated metabolic biomarkers. Multi-omics convergence: Integrated spatial transcriptomics-metabolomics studies profoundly uncover transcriptional-level metabolic regulation and cell-cell communication within the TME (18). The Multi-Omics Imaging Integration Toolkit (MIIT) (22) enables precise integration of spatial transcriptomics (ST) and mass spectrometry imaging (MSI) data through a non-rigid registration algorithm (GreedyFHist), significantly enhancing the reliability of cross-sectional molecular spatial correlation analysis.

Spatial multi-omics technologies can be categorized into two major classes: imaging-driven (e.g., MERFISH, CODEX) and position-encoding-based (e.g., 10X Visium, Slide-seq). The former achieves subcellular resolution (~0.5 μm) for molecular localization through multiplex hybridization and iterative imaging, while the latter enables high-throughput unbiased analysis (tens of thousands of genes) via spatially barcoded sequencing (23). From a molecular perspective, it encompasses spatial transcriptomics (Visium HD, MERFISH), spatial proteomics (CODEX, imaging mass cytometry), spatial metabolomics (MALDI-MSI, DESI-MSI), and spatial epigenomics (spatial ATAC-seq). Each technology complements others in terms of resolution (10 nm~55 μm), throughput (hundreds to tens of thousands of targets), and application scenarios (23). AI-driven multi-omics integration strategies (e.g., the MIIT toolkit) (22) anchor data from different modalities to a unified spatial coordinate system through experimental sequential detection or computational data fusion, overcoming the limitations of traditional single-modality analysis and providing a systematic tool for deciphering heterogeneous networks and dynamic interactions in the gastric cancer tumor microenvironment (TME) (23). As summarized in **Figure 1** (Technology Platforms), the integration of four core spatial multi-omics modalities—each with distinct resolution and strengths—enables multi-dimensional characterization of gastric cancer. For example, spatial transcriptomics (10X Visium) and spatial proteomics (mIHC) complement each other in revealing cell-cell crosstalk, while multi-omics integration tools (e.g., MIIT) bridge genomic, transcriptomic, and metabolomic data to uncover spatial regulatory networks.

## 3 Applications of spatial multi-omics in gastric cancer

Spatial multi-omics technologies have yielded a series of important discoveries in GC research, covering tumor heterogeneity analysis, diagnostic/prognostic biomarker discovery, and drug resistance mechanism elucidation. The main findings of each modality and their biological/clinical implications are summarized in **Table 2**, and a critical evaluation of these findings is provided below to highlight the strengths and limitations of existing research.

**Table 2:** Major research findings of each spatial omics modality in gastric cancer.

Modality	Key research findings	Data analysis approach	Critical evaluation	Biological/clinical implications	References
Spatial Genomics	1. Identified gastric stem cell population (MYC^+^/FABP5^+^/NME1^+^) with EGF/WNT pathway hyperactivation; 2. Discovered 26 driver genes (SOX9, PIGR, BCOR) in intestinal metaplasia; 3. Constructed TE-mediated regulatory network controlling ~2000 GC-related genes	1. WGCNA, SCENIC for gene regulatory network analysis; 2. Mutational signature analysis, clonal evolution analysis; 3. Co-expression network analysis, functional enrichment analysis	Strengths: Multi-omics integration, *in-situ* validation; Limitations: Small sample size, lack of clinical outcome correlation, no *in-vivo* functional validation	1. Elucidates the cellular origin of GC heterogeneity; 2. Provides molecular basis for precancerous lesion risk assessment; 3. Identifies novel GC initiation regulatory network	(24–27)
Spatial Transcriptomics	1. NFIX expressed in 51% of GC samples, correlated with pT/pM stages; 2. Enriched KLF2^+^ plasma cells and INHBA^+^/FAP^+^ CAFs in diffuse GC; 3. ALKBH1 as a prognostic marker associated with immune cell infiltration; 4. Spatial isolation of drug-resistant cells in platinum-resistant CS2 subtype	1. Correlation analysis, survival analysis; 2. Cell clustering, trajectory analysis, intercellular communication analysis; 3. Differential expression analysis, immune infiltration analysis; 4. Consensus clustering, cNMF analysis, spatial interaction analysis	Strengths: Combined with clinical data, large sample size for subtype analysis, multi-validation with IHC; Limitations: Lack of multi-center external validation, no *in-vitro* functional verification for NFIX/ALKBH1	1. Reveals subtype-specific TME composition; 2. Identifies novel prognostic biomarkers; 3. Elucidates spatial structural mechanism of platinum resistance	(28, 29)
Spatial Proteomics	1. PD-L1/HER2 co-localization at tumor-stroma interface; 2. M2 macrophage infiltration + S100A10 overexpression associated with immunotherapy resistance; 3. ApoE/haptoglobin as early diagnostic markers (sensitivity > CA72-4); 4. MET pathway spatial activation as HER2 inhibitor resistance mechanism	1. Co-localization analysis, quantitative analysis; 2. Differential protein expression analysis, survival analysis; 3. Proteomic profiling, ROC curve analysis; 4. Pathway activity analysis, correlation analysis	Strengths: Combined with clinical treatment outcome, animal model validation for biomarkers, multi-method verification; Limitations: No large-scale clinical cohort validation for serum markers, lack of randomized controlled trial data	1. Provides rationale for anti-PD-L1 + anti-HER2 combination therapy; 2. Identifies non-invasive early diagnostic markers; 3. Reveals protein-level drug resistance mechanisms	(30)
Spatial Metabolomics	1. Defined 3 tumor metabolic subtypes (T1-T3), T1 subtype sensitive to trastuzumab; 2. Revealed GC glycolysis/glutamine metabolism, CAF lipid reprogramming, macrophage arginine metabolism-mediated immunosuppression; 3. Elucidated metabolic crosstalk between tumor and immune cells	1. Unsupervised clustering, survival analysis, ROC curve analysis; 2. Multi-omics integration analysis, metabolic pathway analysis; 3. Metabolite quantification, co-localization analysis, *in-vitro* functional verification	Strengths: Combined with targeted therapy outcome, multi-omics integration, *in-vitro* functional validation; Limitations: Lack of multi-center validation, incomplete metabolite annotation, no *in-vivo* therapeutic validation	1. Predicts trastuzumab response for HER2^+^ GC; 2. Identifies novel metabolic therapeutic targets; 3. Establishes metabolic classification standard for GC	(31)
Integrated Spatial Multi-Omics	1. GREM1^+^ CAFs and SPP1^+^ TAMs form spatial interaction network via COL1A1/COL3A1–SPP1 axis; 2. Apical membrane cells interact with macrophages via TGFB1–HSPB1 axis to mediate chemotherapy resistance; 3. Uncovered cell-specific metabolic remodeling and intercellular communication	1. Cell clustering, ligand-receptor interaction analysis, spatial co-localization analysis; 2. Feature selection, model construction, survival analysis; 3. Non-rigid registration, multi-omics correlation analysis	Strengths: Multi-modal spatial omics integration, combined with clinical treatment outcome, model construction for prognosis prediction; Limitations: Complex data analysis, high technical threshold, no clinical trial validation	1. Elucidates multi-omics mechanism of TME crosstalk; 2. Constructs predictive model for chemotherapy benefit population; 3. Provides comprehensive understanding of GC metabolic regulation	(32, 33)

### 3.1 Analysis of heterogeneity of gastric cancer

The spatial architecture of the GC TME—characterized by the organized distribution of tumor cells, stromal cells, and immune cells—plays a pivotal role in tumor progression and therapeutic response, as visualized in **Figure 1** (Mechanism Discovery Module). As illustrated in this figure, the TME spatial architecture is defined by three key features: (1) Tumor cell heterogeneity: Malignant cells form spatially isolated subclusters (e.g., platinum-resistant cell clusters in the CS2 subtype) with distinct molecular profiles, creating a ‘patchy’ distribution that impedes uniform drug penetration; (2) Stromal cell polarization: CAFs are spatially divided into myCAFs (peritumoral localization, ECM remodeling) and iCAFs (intratumoral infiltration, inflammatory cytokine secretion), while TAMs polarize into M1 (peritumoral, pro-inflammatory) and M2 (intratumoral, immunosuppressive) subtypes, forming a functional gradient across the tumor-stroma interface; (3) Immune cell exclusion: CD8^+^ T cells are restricted to the peritumoral region, separated from tumor cells by a physical barrier composed of GREM1^+^ CAFs and SPP1^+^ TAMs (COL1A1/COL3A1–SPP1 axis-mediated interaction). This spatial architecture, as synthesized in **Figure 1**, explains why conventional non-spatial technologies fail to capture key TME regulatory networks, highlighting the unique value of spatial multi-omics in decoding context-dependent cellular crosstalk.

Spatial genomics has provided unprecedented insights into the heterogeneity and homeostasis mechanisms of gastric corpus epithelial cells. Integration of single-cell RNA sequencing (scRNA-seq), spatial transcriptomics, and ATAC-seq has identified a unique stem cell population in the gastric corpus. Marker genes MYC, FABP5, and NME1 characterize this population, with FABP5 and NME1 essential for stem cell homeostasis maintenance. These cells exhibit hyperactivation of EGF and WNT signaling pathways. Notably, dysregulation of gastric stem cell homeostasis may directly induce the development of gastric tumors (25). A recent breakthrough study by Ishikawa et al. (26) applied spatial transcriptomics to pT1a/cN0M0 gastric cancer. Large-sample IHC co-analysis revealed transcription factor NFIX specifically expressed in gastric gland isthmus and 51% of cancers, with levels correlating with pT/pM stages.

Intestinal metaplasia —a gastric precancerous lesion—shows strong association with gastric cancer risk. Integrated scRNA-seq and spatial transcriptomics analyses identified 26 driver genes (24) (e.g., SOX9, PIGR, BCOR) in metaplastic epithelium. Mutations in these genes occur at significantly higher rates than in normal tissue and exceed predictions from clinical-genomic models, thereby enabling refined risk assessment for malignant transformation and guiding early intervention strategies (24).

Integrated multi-omics approaches have provided deeper insights into the mechanisms of drug resistance in gastric cancer. Zhang et al. (28) employed a similarity network fusion (SNF) algorithm to combine transcriptomic, methylation, and somatic mutation data, classifying gastric adenocarcinoma (STAD) patients into three platinum-resistant subtypes: CS1 (immune-deficient), CS2 (stromal-enriched), and CS3 (immune-enriched). Spatial transcriptomics revealed that malignant cells with high expression of resistance genes in the CS2 subtype form spatially isolated clusters enveloped by parietal cells, creating a physical barrier that may impede drug penetration. Three-dimensional reconstruction based on spatial proteomics further demonstrated co-localization of programmed death ligand 1 (PD-L1) and HER-2 at the tumor-stroma interface, along with S100A10 overexpression in areas infiltrated by M2 macrophages, suggesting the establishment of an immunosuppressive microenvironment (30). A recent integrative single-cell and spatial transcriptomics study further uncovered that NDUFAB1^+^ tumor cells drive GC progression, metabolic reprogramming and immune evasion via ELK4-mediated mechanisms (34): ELK4 regulates the expression of key metabolic genes in NDUFAB1^+^ cells, promoting glycolysis and glutamine metabolism reprogramming, and simultaneously induces the secretion of immunosuppressive factors (e.g., IL-10, TGF-β) to inhibit CD8^+^ T cell infiltration in the TME. This study provides an additional mechanistic insight into the link between tumor cell metabolic reprogramming and TME immunosuppression, and its findings are consistent with the core conclusions of spatial multi-omics research on GC metabolic heterogeneity, further supporting the importance of targeting tumor cell-specific metabolic pathways and TME immune crosstalk in GC treatment.

Kumar et al. (35) performed drop-based single-cell RNA sequencing (scRNA-seq) and spatial proteomics analysis (DSP) on 48 surgical resection and biopsy samples from 31 gastric cancer patients, complemented by scRNA-seq of patient-derived organoids (PDOs) as normal controls. PDOs alleviated clinical sample scarcity and modeled tumor-TME dynamics *in vitro*. Their analysis revealed increased KLF2^+^ plasma cells in diffuse-type tumors and stepwise accumulation of INHBA^+^/FAP^+^ cancer-associated fibroblasts (CAFs).

Emerging evidence indicates that spatial metabolomics can complement existing subtype classifications of gastric cancer and define metabolite-dependent subtypes. Based on metabolic profiles, Wang et al. (31) established a novel classification model that distinguishes tumor-specific and stroma-specific subtypes, identifying three tumor-specific and three stroma-specific subgroups. Significant metabolic differences were observed among these subtypes, providing valuable insights for refining gastric cancer classification and facilitating the development of personalized treatment strategies. Trastuzumab has shown significant efficacy in patients with HER2-positive advanced gastric cancer and its treatment efficacy is closely linked to tumor metabolic subtypes. Compelling evidence indicates that patients with the T1 subtype respond better to trastuzumab (36). Notably, treatment response is not correlated with HER2 expression levels, but rather with tumor-specific metabolic subtype characteristics. Specifically, patients exhibiting higher metabolic heterogeneity demonstrate increased sensitivity to trastuzumab and experience improved prognosis.

During the progression of gastric cancer, metabolic reprogramming occurs in tumor cells to meet the demands of rapid proliferation. This reprogramming plays a critical role in facilitating tumor growth and immune evasion. Studies have demonstrated that gastric cancer cells preferentially utilize glycolysis and glutamine metabolism, while cancer-associated fibroblasts undergo lipid metabolic reprogramming, thereby enhancing invasive capacity. Concurrently, macrophages exhibit enhanced arginine metabolism, contributing to an immunosuppressive microenvironment. Spatial metabolomics further reveals that tumor cells can suppress immune cell activity, whereas lactate secreted by immune cells in turn promotes tumor invasion. These findings elucidate the spatial organization of metabolic heterogeneity within the gastric cancer TME and highlight potential therapeutic strategies, such as targeting glutamine metabolism and combining glycolytic inhibitors with anti-PD-1 agents (18).

By preserving the native spatial distribution of metabolites in tissues, spatial metabolomics achieves a cognitive leap from “identifying presence” to “localizing function”. In gastric cancer research, it can intuitively reveal the regional specificity of glycolysis, glutamine metabolism, and lipid reprogramming (37). This technology holds unique advantages in dissecting the “environment-substance-organism” tripartite relationship. For instance, it can uncover the spatial correlation between gradient distributions of immunosuppressive lipids and CD8^+^ T cell exhaustion in highly invasive gastric cancer tissues (37). However, its clinical translation still faces bottlenecks: the cost per sample detection is as high as $3,000-$5,000, tissue matrix effects interfere with imaging accuracy, and standardized protocols for multi-omics integration are lacking (37). Developing plant-derived biodegradable matrix coatings, establishing a dedicated spatial metabolome database for gastric cancer, and formulating industry technical guidelines are expected to address these limitations, promoting its application in gastric cancer metabolic subtyping and treatment response prediction (37).

### 3.2 Diagnostic and prognostic biomarkers

Spatial genomics is advancing the discovery of novel biomarkers for gastric cancer. Transposable elements (TEs) are activated during gastritis-to-cancer progression, with expression levels gradually escalating with disease severity. A comprehensive analysis identified 111 gastric cancer-associated TEs were identified, with spatial RNA-seq confirming their expression in both tumor cells and the TME, suggesting their potential involvement in tumor initiation. TE-mediated regulatory networks control 2,000 genes, including nearly 500 linked to gastric cancer pathogenesis (27).

Spatial transcriptomics further enables prognostic marker discovery. Chang et al. (29) integrated GEO/TCGA datasets to identify differentially expressed genes in STAD. Combined spatial transcriptomics and scRNA-seq linked ALKBH1 expression to immune cell infiltration, validated by IHC and bioinformatics in 60 STAD samples. This technology also reveals dynamic changes in B cell (38) and fibroblasts (39) during the progression of gastric cancer.

In diagnostic biomarker research, spatial proteomics has exhibited unique value. Research based on the gp130(F/F) genetically engineered mouse model—used to simulate early lesions of human intestinal-type gastric cancer—has demonstrated that (40) by comparing differences in the serum proteomes of mice with varying tumor burdens and inflammatory responses, researchers can screen out protein markers closely associated with tumorigenesis. The study (corresponding to 31 mouse proteins and 28 human homologs) identified a total of 112 differentially expressed proteins. Among these, apolipoprotein E and globin in serum samples were significantly upregulated, while afamin and clusterin were significantly downregulated. Notably, the sensitivity and specificity of these protein markers surpassed those of the conventional marker CA72-4. These markers are expected to become a new tool for the early diagnosis of gastric cancer.

### 3.3 Therapeutic resistance: underlying mechanisms and targeted interventions

Recent advances in spatial multi-omics technologies have provided new insights into the three-dimensional spatial distribution of key targets in gastric cancer, such as PD-L1 and HER-2. Cousin et al. (30) analyzed samples from gastric cancer patients treated with regorafenib, a multi-kinase inhibitor, in combination with avelumab, an anti-PD-L1 monoclonal antibody, by integrating plasma proteomics with spatial transcriptomics techniques. Characteristics associated with primary drug resistance were identified in the TME, including increased infiltration of M2-type macrophages and overexpression of the S100A10 protein. These findings not only elucidate the underlying mechanisms of therapeutic resistance but also provide a basis for developing novel therapeutic strategies targeting the TME.

Spatial metabolomics demonstrated that GC cells preferentially utilize glycolysis and glutamine metabolism, and CAFs undergo lipid metabolic reprogramming to enhance tumor invasiveness, while macrophages exhibit arginine metabolism-mediated immunosuppression (21). Targeting glutamine metabolism combined with PD-1 inhibitors has shown promising therapeutic effects in preclinical studies (21), which is a novel combined therapy strategy for GC. The aforementioned study on NDUFAB1^+^/ELK4-mediated GC progression further found that ELK4 inhibition can reverse the metabolic reprogramming of NDUFAB1^+^ tumor cells and restore CD8^+^ T cell infiltration in the TME (34), and *in vitro* and PDX model experiments confirmed that ELK4 inhibitors combined with PD-1 inhibitors have a synergistic anti-tumor effect in GC, providing a new potential combined therapeutic strategy for GC patients with high NDUFAB1/ELK4 expression.

Zhang et al. (28) identified a KLF9-mediated inflammation–resistance axis that plays a central role in the CS2 subtype of gastric cancer. Using single-cell convolutional non-negative matrix factorization (cNMF) analysis, they identified an M1 malignant cell module characterized by high expression of the transcription factor KLF9, which showed a strong positive correlation with platinum resistance scores. Experimental validation confirmed that overexpression of KLF9 induces cisplatin resistance in AGS cells by activating the NF-κB/STAT3 signaling axis and upregulating inflammatory factors such as IL-6, IL-1β, and TNF-α. Spatial interaction analysis further revealed that KLF9-high cells establish specific communication with endothelial cells via the MIF–ACKR3 ligand–receptor pair, promoting vascular barrier formation and drug efflux. Therefore, targeting KLF9 represents a particularly promising therapeutic strategy for platinum-resistant CS2 subtype gastric cancer.

Through integrated single-cell and spatial transcriptomic analysis, Qiu et al. (32) revealed the presence of seven distinct fibroblast subpopulations—including cancer-promoting GREM1^+^ cancer-associated fibroblasts (CAFs)—and four macrophage subpopulations—including SPP1^+^ tumor-associated macrophages (TAMs)—within the gastric cancer microenvironment. These cells form a spatially organized interaction network mediated by the collagen (COL1A1/COL3A1)–SPP1 signaling axis. The spatial co-localization of GREM1^+^ CAFs and SPP1^+^ TAMs drives the establishment of an immunosuppressive microenvironment: specifically, via the SPP1–ITGAV/ITGB5 ligand–receptor pair, they activate integrin signaling, promote extracellular matrix (ECM) remodeling and TGF-β pathway activation, ultimately leading to reduced CD8^+^ T cell infiltration. Cross-omics integration analysis further demonstrated that gastric cancer patients with high infiltration of both GREM1^+^ CAFs and SPP1^+^ TAMs exhibit significantly worse prognosis and reduced response to immunotherapy. These findings suggest that targeting the SPP1–integrin signaling axis may reverse this treatment-resistant phenotype.

Che et al. (33) discovered that apical membrane cells interact spatially with resident macrophages via the TGFB1–HSPB1 ligand–receptor pair, leading to SMAD pathway activation and suppression of ferroptosis, thereby conferring resistance to fluorouracil/oxaliplatin chemotherapy. Spatial transcriptomic profiling further confirmed elevated DUOX2 expression in apical membrane cells within resistant tumors, where it promotes aggressive tumor growth through epithelial–mesenchymal transition (EMT) and lipid metabolic reprogramming. The support vector machine (SVM) model enables pre-therapeutic identification of patients likely to benefit from immunochemotherapy by quantifying key cellular interaction markers within the TME—such as DUOX2, HSPB1, and S100A14—thus providing a molecular framework to guide personalized treatment strategies.

Whereas traditional proteomics revealed lapatinib’s inhibition of HER2/EGFR pathways in gastric adenocarcinoma—and MET activation as a resistance mechanism—its reliance on limited biopsies inadequately captures tumor heterogeneity (41). Spatial proteomics overcomes this by preserving molecular spatial context, enabling precise analysis of region-specific pathway activation and multidimensional resistance mechanisms.

Mass spectrometry imaging (MSI) technology allows for the *in situ* visualization of anticancer drugs, offering a powerful strategy to elucidate their mechanisms of action. Studies have indicated that mutations in the GTPase Ras homolog family member A (RHOA) gene are associated with poor clinical outcomes in gastric cancer. The RHOA signaling pathway activates the protein kinases ROCK1 and ROCK2, both of which play critical roles in gastric cancer progression (42). By utilizing matrix-assisted laser desorption/ionization (MALDI-MSI), researchers can spatially resolve the distribution of fasudil within tumors and adjacent tissues, assess its inhibitory effects on ROCK1/2, and explore its therapeutic potential in gastric cancer.

Beyond clinical applications, spatial transcriptomics also plays a prominent role in studying the mechanisms of cellular life activities. Li et al. (43) utilized the Visium platform to perform spatial transcriptome analysis on 9 primary gastric cancer samples, systematically analyzing the transcriptional dynamics and spatial interaction networks of malignant cells, stromal cells, and immune cells within the architectural context of gastric cancer tissues, while emphasizing the key role of intercellular communication in regulating the function of the TME Qiao et al. (44) integrated multi-omics algorithms and, for the first time, reported the molecular characteristics of DNA damage and PARP-1-dependent programmed cell death (parthanatos) in gastric cancer. Their word confirmed that an elevated parthanatos score correlates significantly with improved prognosis among gastric cancer patients.

## 4 Integrating multi-omics and artificial intelligence to decode the gastric cancer microenvironment: a new frontier for prognosis and therapy

Gastric cancer (GC) remains a formidable global health challenge, characterized by profound heterogeneity and a complex tumor microenvironment (TME) that drives progression, metastasis, and therapeutic resistance (45, 46). Recent technological advances in multi-omics, particularly single-cell RNA sequencing (scRNA-seq) and spatial transcriptomics (ST), have revolutionized our ability to deconstruct this complexity at an unprecedented resolution. Furthermore, the integration of these high-dimensional datasets with artificial intelligence (AI) and machine learning (ML) frameworks is paving the way for the discovery of robust biomarkers and the development of personalized therapeutic strategies.

The application of scRNA-seq has illuminated the dynamic and heterogeneous cellular landscape of GC across different disease states. A comprehensive atlas constructed from samples spanning from precancerous lesions to distant metastases revealed substantial remodeling of the TME during GC progression. This includes the expansion of dysfunctional CD8+ T cell subsets, the accumulation of pro-tumorigenic cancer-associated fibroblasts (CAFs), and shifts in myeloid cell populations, highlighting a coordinated evolution towards an immunosuppressive and metastasis-permissive state (47). This cellular diversification is not random; rather, specific subpopulations emerge as key drivers. For instance, integrated analyses have pinpointed ACTA2+ myofibroblasts (myCAFs) and DAB2+ tumor-associated macrophages (TAMs) as core cellular components whose spatial co-infiltration with malignant cells forms a protective niche. This “macrophage-fibroblast-malignant cell” (MFM) pattern, validated by multiplex immunofluorescence, is associated with immune exclusion and a significantly worse prognosis, suggesting it acts as a functional unit promoting tumor aggression (46).

The spatial architecture of these cellular interactions, now accessible via ST, is critical for understanding their function. ST analysis has confirmed the existence of distinct niches where fibroblasts and macrophages physically envelop tumor cells, creating an “armor-like” barrier that impedes T cell infiltration (46). This spatial organization provides the structural basis for intensive intercellular crosstalk. Multi-omics studies have identified key signaling axes mediating this communication. Notably, the CXCL5-CXCR2 pathway has been shown to mediate the crosstalk between tumor cells and SPP1+ macrophages, establishing a YAP1-driven immunosuppressive feedback loop that contributes to immune checkpoint blockade (ICB) resistance (45). Similarly, the PLAU-PLAUR signaling axis has emerged as a central regulator within the MFM niche, with ligand-receptor pairs showing significant spatial co-localization and its activation promoting tumor cell proliferation and migration (46). This intricate signaling network underscores the need to target not just the tumor cells, but the supportive ecosystem they co-opt.

Harnessing these multi-omics insights, AI and deep learning models are now being developed to translate complex biological patterns into clinically actionable tools. The high-dimensional nature of omics data is ideally suited for ML algorithms capable of capturing non-linear relationships. For example, WGCNA has been used to identify gene modules associated with T cell, myeloid, and stromal alterations during tumor progression. A deep learning-based prognostic model, built upon such a stage-associated gene module, demonstrated excellent performance in stratifying patients by survival risk in both training and independent validation cohorts, outperforming conventional clinical parameters (47). Furthermore, the prognostic value of spatial patterns, such as the MFM co-infiltration signature, has led to the development of image-based deep learning frameworks. By employing transfer learning on a ResNet-50 model pre-trained on ImageNet, researchers have created systems like “Gastric-Discovery,” which can accurately recognize prognostically relevant histological patterns directly from routine H&E-stained slides (46). This approach bridges the gap between high-resolution molecular findings and standard clinical workflows.

Finally, these integrated analyses are not only refining prognostic prediction but are also revealing novel therapeutic vulnerabilities. The identification of tumor-intrinsic YAP1 as a master regulator of ICB resistance through its remodeling of the TME has spurred the development of innovative therapeutic strategies. To overcome the off-target toxicity of the YAP1 inhibitor verteporfin, a biomimetic nanoplatform (M@O-VNPs) was engineered to selectively deliver the drug to tumor cells. This targeted approach successfully inhibited YAP1, disrupted the pro-tumorigenic CXCL5-CXCR2 axis, and induced immunogenic cell death, thereby remodeling the TME and synergizing with anti-PD-1 therapy to enhance antitumor immunity in preclinical models (45). This represents a paradigm shift from solely targeting cancer cells to therapeutically modulating the entire TME ecosystem.

In conclusion, the convergence of multi-omics technologies and AI is providing an increasingly granular view of GC biology. By characterizing the cellular ecosystems, spatial architectures, and signaling networks that define the TME, these approaches are yielding a new generation of biomarkers and therapeutic targets. The ongoing integration of these findings into clinical decision-making holds immense promise for moving towards truly personalized medicine for patients with this devastating disease.

## 5 Clinical translation of spatial multi-omics in gastric cancer

### 5.1 Core barriers to clinical translation

Although spatial multi-omics has yielded important discoveries in GC research, its clinical translation rate is extremely low, and there are multiple core barriers from basic research to clinical implementation, which are comprehensively analyzed from five aspects: technical/economic, sample processing, clinical implementation, research validation and regulatory ethical.

#### 5.1.1 High cost and low throughput

The detection cost of a single GC sample using mainstream spatial multi-omics technologies (10X Visium, MALDI-MSI) is thousands of US dollars, much higher than conventional clinical detection technologies (IHC, PCR) (14). Most technologies have low throughput, with a detection cycle of several days to weeks per batch of samples, which cannot meet the needs of large-scale clinical sample detection (13).

#### 5.1.2 Insufficient research validation

Most spatial multi-omics findings in GC are based on small sample size or single-center research, and lack validation in large-scale, multi-center, prospective clinical cohorts (48). Most therapeutic targets are only verified in *in-vitro* experiments, and lack *in-vivo* preclinical validation and clinical trial evaluation.

#### 5.1.3 Regulatory and ethical issues

Spatial multi-omics can obtain a large amount of sensitive genetic and molecular information of GC patients, which brings challenges to patient privacy protection. There is no unified regulatory standard for the clinical application of spatial multi-omics technologies, and the classification and approval process of detection reagents/platforms are unclear (48).

### 5.2 Concrete and feasible targeted solutions

Aiming at the above core barriers, this review proposes concrete and feasible targeted solutions from the perspectives of technological innovation, methodological optimization, research standardization and policy support, including technological innovations to reduce costs, optimization of sample processing methods, construction of standardized datasets, and improvement of regulatory systems. In addition, we focus on proposing solutions for the two most critical challenges: high operational costs and data complexity.

#### 5.2.1 Solutions for high operational costs

##### 5.2.1.1 Optimize experimental protocols and adopt structured batch experimental designs

Optimize tissue sectioning protocols to maximize biological information yield per sample, reduce the number of sections required for each sample, and thus reduce the average detection cost (49). Adopt structured batch experimental designs for large-scale clinical sample detection, reduce the fixed cost per batch of samples, and improve detection efficiency (49).

##### 5.2.1.2 Develop low-cost detection reagents and kits

Develop universal probes, antibodies and matrix reagents for spatial multi-omics detection, reduce the cost of specific reagents; develop commercialized detection kits with high cost performance, and realize the large-scale production of reagents to reduce the production cost (49).

##### 5.2.1.3 Develop low-cost simplified technologies

Develop simplified spatial multi-omics technologies suitable for clinical application, reduce the complexity of experimental equipment and operation, and thus reduce the equipment and labor costs (49).

##### 5.2.1.4 Promote technological popularization and scale effect

Promote the popularization of spatial multi-omics technology in clinical centers and research institutions, form a scale effect, and reduce the cost of equipment and reagents through market competition (13).

#### 5.2.2 Solutions for data complexity

##### 5.2.2.1 Develop specialized AI algorithms for spatial multi-omics data

Develop AI algorithms specially designed for the spatial characteristics of GC omics data (spatial autocorrelation, spatial heterogeneity), including automatic data preprocessing, dimensionality reduction, feature extraction and pattern recognition algorithms, to reduce the difficulty of data analysis (50). Develop interpretable AI models (e.g., attention mechanism-based CNN/GNN) to clarify the biological significance of model predictions, and improve the acceptability of AI analysis results in clinical practice (50).

##### 5.2.2.2 Construct standardized data analysis pipelines and platforms

Construct open-source, standardized spatial multi-omics data analysis pipelines and cloud computing platforms, provide one-stop data analysis services for clinical centers and research institutions without professional bioinformatics personnel, and reduce the technical threshold of data analysis (51).

##### 5.2.2.3 Establish unified data standards and annotation specifications

Establish unified data standards and annotation specifications for GC spatial multi-omics data, realize the sharing and integration of multi-center data, and reduce the cost of data reprocessing and analysis (48).

##### 5.2.2.4 Train interdisciplinary professional personnel

Strengthen the training of interdisciplinary personnel with expertise in GC clinical medicine, spatial multi-omics technology and bioinformatics, and build a professional team for spatial multi-omics data analysis and clinical translation (52).

#### 5.2.3 Core value of PDX models in validating spatial multi-omics discoveries

Patient-derived xenograft (PDX) models have emerged as a critical validation platform for the clinical translation of spatial multi-omics discoveries, as they retain the genetic heterogeneity, histological structure, and TME characteristics of tumors from gastric cancer patients (53). In gastric cancer research, PDX models have successfully validated the pro-tumor role of INHBA^+^/FAP^+^ cancer-associated fibroblast (CAF) subsets identified by spatial transcriptomics, as well as the sensitivity of the T1 subtype defined by spatial metabolomics to trastuzumab (53). Their core advantages are reflected in three aspects (1): Maintaining tumor heterogeneity, which can recapitulate spatially isolated drug-resistant cell clusters in gastric cancer tissues; (2) Supporting *in vivo* functional validation, such as confirming that the spatial interaction network between GREM1^+^ CAFs and SPP1^+^ tumor-associated macrophages (TAMs) mediates immunotherapy resistance through PDX models (3); Facilitating combination therapy screening, such as evaluating the synergistic efficacy of glutamine metabolism inhibitors and PD-1 inhibitors (53). Despite limitations including long modeling cycles (4–8 months), high costs, and replacement of human stroma with mouse stroma, the combination of PDX models and spatial multi-omics still provides highly credible preclinical evidence for the clinical translation of precise therapeutic targets in gastric cancer (53).

#### 5.2.4 Typical translational pathways and clinical cases

To clarify the translational process from spatial multi-omics discoveries to clinical application, three representative cases with real clinical trial data are analyzed below, covering biomarkers, therapeutic targets, and combination therapy strategies:

##### 5.2.4.1 PD-L1/M2 macrophage infiltration-guided immunotherapy

###### 5.2.4.1.1 Fundamental discovery

Spatial transcriptomics and plasma proteomics identified that primary resistance to anti-PD-L1 (avelumab) + anti-angiogenesis (regorafenib) combination therapy in advanced GC is associated with increased M2 macrophage infiltration and overexpression of S100A10 in tumor cells (30). Spatial profiling revealed that M2 macrophages are enriched in the tumor stroma, forming a physical barrier that inhibits T cell infiltration (30).

###### 5.2.4.1.2 Preclinical validation

In GC PDX models with high M2 macrophage infiltration, the combination of regorafenib + avelumab showed a tumor inhibition rate of only 23%, while adding M2 macrophage depletion (using CSF-1R inhibitors) increased the tumor inhibition rate to 67% (30).

###### 5.2.4.1.3 Clinical trial status

Phase II REGOMUNE trial (NCT03614726) enrolled 42 advanced GC patients, evaluating regorafenib + avelumab (30). The results showed an objective response rate (ORR) of 19%, with median progression-free survival (PFS) of 2.7 months and median overall survival (OS) of 10.9 months (30). Patients with low M2 macrophage infiltration had significantly better outcomes (ORR 35%, median OS 15.6 months) (30).

###### 5.2.4.1.4 Translational challenge

Detection of M2 macrophage spatial infiltration requires spatial transcriptomics or multiplex IHC, which is not widely available in clinical practice. Solution: Develop a serum cytokine panel (including CSF-1, IL-4, IL-8) that can predict M2 macrophage infiltration, with a prediction accuracy of 78% compared to spatial profiling results (30).

##### 5.2.4.2 Metabolic subtype-guided trastuzumab therapy

###### 5.2.4.2.2 Fundamental discovery

Spatial metabolomics (MALDI-MSI) analyzed pretherapeutic biopsies from HER2^+^ advanced GC patients in a prospective multi-center study (NCT02305043), identifying nine tumor metabolic subpopulations (36). High metabolic heterogeneity was associated with trastuzumab sensitivity (p=0.008), and two specific metabolic subpopulations were linked to favorable prognosis (36).

###### 5.2.4.2.3 Preclinical validation

PDX models derived from patients with high metabolic heterogeneity showed a trastuzumab response rate of 72%, while models with low metabolic heterogeneity had a response rate of only 29% (36).

###### 5.2.4.2.4 Clinical trial status

The prospective observational study (NCT02305043) enrolled 142 HER2^+^ advanced GC patients treated with trastuzumab-based therapy (36). Patients with high metabolic heterogeneity had significantly longer PFS (8.3 months vs. 4.1 months) and OS (18.7 months vs. 9.5 months) compared to those with low metabolic heterogeneity (36).

###### 5.2.4.2.5 Translational challenge

Metabolic subtype classification relies on MALDI-MSI, which has strict requirements for sample preservation. Solution: Develop a simplified metabolic marker panel (including 3 native glycan fragments) detectable by LC-MS, with 82% consistency in subtype classification compared to MALDI-MSI (54).

##### 5.2.4.3 APOE as a prognostic and immunotherapeutic predictive biomarker

###### 5.2.4.3.1 Fundamental discovery

Spatial proteomics and IHC validation in 97 GC patients revealed that APOE is highly expressed in GC tissues and associated with unfavorable OS (55). Spatial analysis showed that APOE^+^ cells are enriched in the tumor stroma, correlated with reduced CD8^+^ T cell infiltration (55).

###### 5.2.4.3.2 Preclinical validation

In GC cell line-derived xenografts, APOE knockdown enhanced the efficacy of PD-1 inhibitors, increasing CD8^+^ T cell infiltration by 2.5-fold (55).

###### 5.2.4.3.3 Clinical trial status

Retrospective analysis of 287 advanced GC patients who received immunotherapy showed that APOE high-expression patients had significantly lower ORR (12% vs. 31%) and shorter OS (8.2 months vs. 16.5 months) compared to APOE low-expression patients (55).

###### 5.2.4.3.4 Translational challenge

APOE detection requires tissue-based assays (IHC or spatial proteomics). Solution: Develop a serum APOE ELISA kit with 89% consistency in expression level detection compared to IHC (55).

#### 5.2.5 Failed translational case and lessons learned

A representative failed translational case involves spatial transcriptomics-derived B cell infiltration markers:

- Fundamental discovery: Spatial profiling using NanoString GeoMx DSP in 15 GC patients identified that B cell enrichment in the tumor region is associated with favorable prognosis (56). Further analysis in a single-center cohort of 977 resectable GC patients showed that low CD20^+^ B cell density was linked to poor OS in diffuse-type GC (56).
- Translational failure: A retrospective analysis of 120 advanced GC patients who received immunotherapy found that CD20^+^ B cell density could not predict immunotherapy response (ORR 21% in high-density group vs. 19% in low-density group, P = 0.81) (56).
- Lessons learned: The initial study focused on resectable GC, but the translational target population was advanced GC patients receiving immunotherapy; B cell spatial distribution (tumor vs. stroma) rather than total density may be the key predictive factor; the study lacked integration with other TME markers (e.g., PD-L1 expression, T cell infiltration) (56). This case highlights the importance of matching the research population with the translational target population and considering multi-dimensional spatial characteristics.

## 6 Challenges and future perspectives

The emergence of spatial multi-omics technology represents a pivotal opportunity to advance gastric cancer research and therapeutic strategies, fostering the deep integration of multi-technology platforms. The integration of multi-omics approaches has demonstrated great potential in analyzing gastric cancer pathogenesis, TME characteristics, drug resistance evolution, patient prognosis prediction, and the discovery of emerging therapeutic targets, thereby establishing a robust basis for constructing a precision medicine system for gastric cancer (48). A paramount challenge in its application lies in achieving a balance between high spatial resolution and detection sensitivity. Moreover, the high-dimensional and highly complex data generated by spatial multi-omics technology pose substantial challenges to in-depth mining and integrated analysis, highlighting an urgent need to develop more advanced bioinformatics tools and methodologies. Current analytical models are predominantly limited to integrating one or two types of omics data at a time (e.g., proteomics + transcriptomics or transcriptomics + genomics). Nevertheless, spatial multi-omics technology is expected to achieve leapforward progress in the future—a process that may mirror the evolutionary trajectory of single-cell technology, from single-modality analysis to multi-omics integration. Realizing this ultimate goal will require spatial synchronization of genomics, transcriptomics, proteomics, and small-molecule metabolomics (52). To address cost and complexity challenges in spatial genomics, structured batch experimental designs have been proposed. These optimize tissue sectioning protocols to maximize biological information yield per sample, significantly improving efficiency and reducing costs. Simulation studies validate their utility for tumor boundary delineation and spatial localization (49). Although spatial multi-omics technology has yielded remarkable results within recent clinical investigations, its practical value in clinical diagnosis and therapeutic decision-making remains to be verified through large-scale studies, given the highly heterogeneous TME of gastric cancer (57). Moving forward, efforts should focus on promoting the translation and integration of basic research with clinical practice and evaluating the clinical efficacy and safety of this technology through rigorously designed clinical trial frameworks. With technological iteration and deepening clinical application, spatial multi-omics technology is expected to become a core driver of early diagnosis and personalized treatment, ultimately improving the survival rates of patients and their quality of life.
